# The Rebel Body: The Subversive Meanings of Illness

**DOI:** 10.1007/s11013-025-09968-7

**Published:** 2026-03-11

**Authors:** Nancy Scheper-Hughes, Julia Vorhölter, Amand-Gabriel Führer

**Affiliations:** 1 University of California, Berkeley, Berkeley, USA; 2 Max Planck Institute for Social Anthropology, Halle, Germany; 3 Martin Luther University Halle-Wittenberg, Halle, Germany

## Abstract

Nancy Scheper-Hughes’s and Margaret Lock’s (1987) article on the “the mindful body,” in which they introduce their framework of three interconnected bodies (individual, social, and the body politic), has shaped debates in medical anthropology over the last three decades and, as Yates-Doerr (2017: 142) puts it, represented “*a zeitgeist for the field*” (italics in original). Scheper-Hughes’s related, but more politicized idea of the “rebel body,” however—which she sketches in the following reprint—has not yet entered mainstream debates.

Originally published only in print in the *Traditional Acupuncture Society Journal* (Scheper-Hughes, 1991), the article conceptualizes the rebel body as one that “refuse[s] the demand to suffer quietly” and thereby reveals and challenges political etiologies of illness. We discovered the article in our preparation of the special issue (see Führer and Vorhölter, 2025) and found it to be extremely valuable for our reflections on liberation medicine—and surprisingly timely. The article offers a compelling analysis of the political causes and potentials of illness, of pain and its demand for recognition, and of the power of refusal. While some of these themes have since been prominently discussed in more recent scholarship (see e.g., Buchbinder, 2015; Hamdy, 2008; Rose Hunt, 2016; McGranahan, 2016; Simpson, 2014), the conceptualization of the rebel body remains provocative and relevant to contemporary debates in and on medicine. By republishing this article here, we hope to make it accessible to a new generation of scholars, practitioners, patients, and activists and hereby further their aspirations toward an understanding of medicine as a form of everyday resistance.

The article begins with a survey of anthropological understandings of and debates on the body, embodiment, and somatization. Based on her own fieldwork in North-Western Brazil, and using the framework of the “three bodies,” Scheper-Hughes reflects on the interrelations between the individual body, the social body, and the body politic. Through a reworking of established notions of illness, suffering, and healing, she proposes to understand illness as a form of bodily praxis that can be read as an expression of protest and rebellion to unequal and unjust social and political orders. Thus read, the moment of illness carries the potential for radical reflection and subsequent action, which medicine as well as society can either mute through biomedical cooptation or respond to with engagement in political therapy.

We are republishing the article with the kind permission of the British Acupuncture Council. The text has been lightly edited and this introductory note/abstract has been added by Amand-Gabriel Führer and Julia Vorhölter. We have added references that were missing in the original article and have removed those that were not mentioned in the text. Furthermore, we have included the works that we cite in this introductory note/abstract in the reference section. The article in the present form is reprinted with the permission of the author.

## Introduction: The Body in Anthropology

My point of departure is an early and highly schematic essay by Mauss (1938), “The Notion of Body Techniques,” in which Mauss reflects on the body as the original medium of human praxis. “The body,” he writes, “is the first and the most natural tool of man.” While we might, today, take exception with Mauss’ notion of “the natural” and with his generic use of “man,” we cannot deny the phenomenological insight on which his assertion is based: the idea that we both are and have bodies and that we *think* and *act* with it and through our corporeal selves. Rather than an inert, dead weight attached to a lively and nomadic “mind,” we have come to understand the body as an active, communicative agent of the self, imbued with its own wisdom, intentionality and language.

Embodiment as a paradigm in medical anthropology concerns the ways that people come to “inhabit” their bodies so that these become, in every sense of the term, “habituated.” This is a play on Mauss’ original meaning of “habitus” (a term later appropriated by Bourdieu, 1977) by which Mauss meant all the acquired habits and somatic tactics that represent the “cultural arts” of using and being in the body (and in the world). From the phenomenological perspective of embodiment, used by some critical and interpretive medical anthropologists, illness, disability and other forms of human suffering are just such habituated bodily expressions of dynamic social and political relations. Sickness is more than an unfortunate brush with nature, and more than something that “just happens” to people; it is something that people *do* in uniquely human and creative ways. Illness is a form of bodily praxis.

### Against—And Beyond—Somatization

It is to the secret language of the body—illness symptoms in particular—that my remarks are addressed. They may be taken as an attempt to supersede the traditional idiom of “somatization” that is so popular in medical anthropological circles today and which, I suggest, continues to feed upon the Cartesian (later Freudian and psychoanalytic) disregard for the body as an unconscious and pre-reflexive, but nonetheless stubborn and willful, agent of the mindful self.

Here I will try to recuperate the body in medical anthropological and psychoanalytic discourse by focusing on the body’s “wisdom,” its intentionality and purposefulness. I will focus on the production of chaotic and unruly symptoms—symptoms that disrespect and that breach the boundaries between nature and culture, mind and body, individual and social bodies. The body, it would seem, is “naturally subversive.” It refuses to conform to our Western epistemology with its neat oppositions and dualisms, its radical individualism, its reductionist materialisms.

At first, medical anthropologists were content to work within the limited psychiatric framework of Eisenberg’s (1977) and Kleinman’s (1980) conceptual distinction between *illness* as perceived by the patient and *disease* as diagnosed by the physician. The patient’s experience of her sickness was understood as an important “modifier” of the otherwise reified reductionist and universal categories of biomedical diagnosis and nosology. Later, due to the influential writings of critical interpretive medical anthropologists, such as Allan Young, Ronald Frankenberg, Jean Comaroff, Margaret Lock, and Michael Taussig, among others, *illness* was rescued from the individualist, ethnocentric domain of doctor–patient relations and understood in more collectivist and social terms as personal or cultural narratives, as drama and performance, and as both individual ceremonies and collective rituals of bodily and social reform.

Two important critically interpretive studies of illness and the mindful body are Gelya Frank’s article “On Embodiment” (published in *Culture, Medicine and Psychiatry* in 1986) and Michael Taussig’s article, “Reification and Consciousness of the Patient” (published in 1980 in *Social Science and Medicine*). Frank presents the case of Diane DeVries, a young woman with congenital quadrilateral limb deficiencies; in other words, she is without arms and legs. Nonetheless, there is an intactness to Diane’s description of her body which, while deviant and deficient in terms of the dominant culture, is natural and native to her own experience. She stands at odds with the medical professions’ technicalized, medicalized view of her condition. She is healthily “embodied,” identifying herself with the mythic beauty of the Venus de Milo, and she experiences no “phantom limbs” where the medical and “helping” professions assume they ought to be. Diane refuses to wear the prescribed prosthetic devices. And why *should* she? She already has a whole and fully habituated body/self.

Taussig’s paper on medical dimensions of reification draws its inspiration from Marx’s analysis of the commodity, which Lukács applied to an interpretation of capitalist culture’s mode of turning social relations into *things*. Taussig explores the signs and symptoms of one woman’s perplexing “disease”—an idiopathic, but fatal, case of severe muscle inflammation called polymyositis—which he shows to reveal as much about the malfunctioning (and contradictions) of social and medical relations as about the malfunctioning of the woman’s body.

While the body can be used, as symbolic anthropologists have indicated, to express a sense of belonging and affirmation, it can also be used to express negative, conflictual sentiments—feelings of distress, alienation, frustration, anger, resentment, sadness and loss. These two modes of bodily expression exist in a dialectical relationship, an expression of the tensions between belonging and alienation that characterize human social life everywhere. In social systems characterized by a great deal of institutionalized inequality and class, racial or gender exploitation, feelings of oppression, frustration, and submerged rage are common, but disallowed, social sentiments. In the following section, I will further elaborate on different expressions of antagonistic feeling and outline the role of illness as a form of subaltern resistance.

## The Sickness Strategy: Forms of Subaltern Resistance

The most immediate way of dealing with feelings of anger and frustration is through direct physical and verbal aggression. For obvious reasons, however, the use of direct confrontation by the aggrieved and submerged sectors of society against their oppressors is only rarely resorted to and is more notable, historically, for its absence. Subversive political activities are dangerous for most people in the world today.

James Scott has pointed out that most subordinate classes throughout history have rarely been afforded the “luxury of open, organized political activity” (1985). This argument can readily be extended to the situation of the majority of women. Nonetheless, those who are relatively powerless *and* aggrieved do put up a remarkable assortment of resistances, including, as Scott pointed out “foot dragging, dissimulation, desertion, false compliance, pilfering, feigned ignorance, slander, arson, sabotage, and so on” (1985).

### Witchcraft and Sorcery

To these, critically interpretive medical anthropologists would add those kinds of institutionalized practices that appear with great frequency in our writings: accusations of witchcraft, sorcery, or the evil eye, gossip, the use of trance, and organized rituals or reversal and fantasy play.

As Mullings (1984) noted with respect to her research in Ghana, witchcraft and sorcery accusations are used widely to explain human misfortune and they function as “metaphors for social relationships.” Within the context of a rapidly industrializing West African urban society, these traditional metaphors are increasingly invoked to express and condemn the contradictions in social relations introduced by industrial capitalism. And so, the accusations today focus especially on those who engage in unfair competition, who are seen as “greedy” and “individualist” in their behavior. Such individuals are perceived as making others in the community ill. Mullings is not, of course, suggesting that witchcraft and sorcery are unique to capitalist social and economic formations, but rather that in the context of the increasing “commoditization” of human life in modern Ghana, witchcraft-produced ills and sorcery accusations are an expression of social tensions and of hostilities related to capitalist practices and values.

Indeed, in contrast, Favret-Saada (1977, l989) shows how witchcraft plays a formidable role in the social relations of a *traditional* peasant economy in the Bocage of western France. In this small, tense, closed familistic community, male heads of households often fall victim to illness through bewitchment. Here, witchcraft serves as a projective institution, expressing the farmer’s guilt feelings concerning his own legally and culturally sanctioned acts of “violence” against relatives that are entailed in the acquisition, through inheritance, of a farm household. Farm inheritance means that one’s own siblings have been dispossessed and that as head of household one will exploit the free labor of a great many close kin—wife, children, unmarried, and disinherited kin. Techniques of “unbewitching,” then, serve to remove the source and focus of perceived hostility and resentment elsewhere, to distant neighbors (who become the key suspects), to those, in fact, with whom there is virtually nothing at stake. And as a “remedial institution,” unbewitching allows the farmer to go about his business free of guilt and fears of reprisals against him.

### Rituals of Reversal

Fantasy play offers aggrieved people another way of circumventing or managing conflict and contradictions that often beset social relations in societies characterized by class or gender inequality. One genre of institutionalized fantasy play found worldwide—through which men and women have expressed their frustration and longings—are the many so-called rituals of reversal and travesty such as the New Guinea gender-crossing Naven ceremony (Bateson, 1958), the Indian feast of love, Holi (Marriot, 1966), and Brazilian carnival (DaMatta, 1983; Parker, 1990; Scheper-Hughes, 1988a).

Common to all these rituals is an inversion of the normative social order and of the moral economy that governs behavior in everyday life. Thrown into the upside down topsy-turvy, carnivalesque atmosphere of license and laughter that these pageants allow, a space is created for the weak, the humbled, the oppressed, and the marginal to take center stage, to wield albeit momentarily, symbolic dominance. Such events offer permission to protest the arbitrary rules that define the “common sense” world, and moreover, permission to deviate from social norms which may be experienced as oppressive: males dress as females; wives publicly ridicule and berate their husbands; peasants mock landowners; and sexuality is displayed rather than hidden.

In all, creative chaos and liminality replace structure and order. Travesty, the ribald, the degraded, and the grotesque are elevated (Bakhtin, 1968). While the potentially revolutionary or socially conservative character of these rituals has long been discussed and heatedly debated (see Gluckman, 1955; Babcock, 1978; Hobsbawm, 1965; Illich, 1982; Davis, 1975; Ladurie, 1980), there has been virtually no examination of the essentially therapeutic nature and functions of these “rituals of complaint,” as we shall henceforth refer to them, for the poor and marginalized individuals who partake in them with such evident enjoyment.

Impoverished Brazilians from the drought-ridden and semi-feudal north-east region to the shanty-towns of Rio and Sao Paulo can spend as much as a quarter of their annual cash fund in the preparations for carnival. While it is all too easy to criticize such behavior as short-sighted and improvident, the psychological and biophysiological benefits of carnival play, travesty, and of solidarity forged through the organization of Brazilian “Samba Schools” have yet to be seriously considered.

### Physical Illness and Distress

Likewise, some forms of physical illness and distress can also be viewed as acts of refusal or of mockery, as a form of protest (albeit not fully conscious) against oppressive social roles and ideologies. Of all the various options for expressing dissent and defiance, the use of illness is perhaps one of the most common, yet also one of the most problematic. We are most familiar with those conventional social and psychological studies of trance and spirit possession in which illness is understood as a largely pre-modern and pre-reflexive technique of “secondary gain.” Here, possession sickness is viewed as a manipulative, individualistic strategy of redress used by poor and marginalized people throughout the world, but especially by women (see Lewis, 1971). In the conventional view, spirit possession is a culturally “exotic” form of conversion hysteria that offers a safe, institutionalized space from which the poor, the oppressed and women can communicate their anger, fear, resentment, anxiety, and envy. It is “safe” because the afflicted are not held responsible for what they say or for the considerable *material* demands they make of others; the “possessed” (who are often also the socially dispossessed) are merely the passive agents through which capricious spirits have their way.

There is communicated in these conventional interpretations an unattractive, cowardly, petulant quality in those who would use such “passive aggressive” strategies. It conjures up, as well, the negative, sexist images of Freud’s pampered Viennese women hysterics, and the likes of Elizabeth Barrett Browning and the “lesser” of the famous James siblings, “poor Alice” James (Edel, 1964; Strouse, 1980), querulously commanding the small world around them within the confines of their sick room. In place of Kafka’s “Hunger Artist,” we have the “Sickness Artist.”

But if we begin to reconsider what Scott (l985) called the “massive middle ground” in between unquestioning deference and violent outrage, that is, the complex, sometimes convoluted means through which the most continuously oppressed social groups historically—peasants, slaves, women, and children—have withheld their consent and registered their non-cooperation with the powerful, we can begin to understand the social and *collective* (rather than the “*merely*” personal) dimensions of the sickness strategy. If we start with a notion of the embodied person living out and reacting to his or her assigned place in the social order, then the social origins of many illnesses and much distress and the “sickening” social order come into sharp focus.

### Three Examples of “sickness strategy”

It is then possible, for example, to interpret the incidences of archaic spirit possession on the modern, sanitized shop-floor of multinational microchip factories in Malaysia as recently described by my Berkeley colleague Ong (1987), as part of a complex negotiation of reality in which women factory workers are reacting both to the violation of their traditional cultural identity and to the demanding work conditions by invoking ancestral spirits so as to bring production to a temporary halt. In place of the demeaning clinical notion of “malingering,” or the more conventionally applied notion of “somatization” we can see instead a “sickness strike,” a strategy not too dissimilar to factory “slowdowns,” the so-called Italian strike, that is used wherever open strikes are likely to be catastrophic in terms of permanent dismissal or jail. Comaroff (1985) has made just this equation in her astute analysis of the way South African Tshidi Zionists use the semantics of bodily affliction within the context of religiously sanctioned trance and possession to express and to redress the colonial and historical roots of their contemporary experience as racially oppressed migrant workers.

Or, we might take the case of “School Refusal Syndrome” in contemporary Japan. “School Refusal” is the medical-psychiatric term used to describe those adolescents who will not go to school, who lie mute and immobilized in their beds throughout the day, indifferent to both rewards and punishment, and who are often heavily medicated as well. The conventional interpretation would be that these young people are reacting defensively and pathologically against the overwhelming competitive pressures of the Japanese school system and to the unrealistic aspirations of their parents. But the critical analysis offered by my colleague and frequent collaborator Lock (1986) implicates a much larger social and political concern with the effects of modernization and the loss of a meaningful and cohesive Japanese identity in which the conflict among the school system, parental values, and the culturally constructed strategy of passive resistance used by the angry and rebellious youth is only a small part.

Similarly, the large body of medical anthropological research and writings on nerves—*nevra/nervios/nervos*—can be reinterpreted so as to supersede the arid and conventional view of dissatisfied, passive–aggressive women offering up a litany of hysterical complaints, with a more accurate and positive view of nervous symptoms as bodily idioms for registering protest and for negotiating power relations.

## Somatization and Somatic Cultures

In many of our recent individual and collaborative writings, Margaret Lock and I (Scheper-Hughes & Lock, 1986, 1987a, b, 1990) have been trying to rescue, recuperate, and politicize the disparaging and stigmatizing concept of “somatization” which plays such a large role in the lives of our subjects (in Japan and in Brazil as well as in the USA) and in the medical anthropology literature generally. To some degree, these writings were an effort to dislodge the earlier and influential writings of Arthur Kleinman who had initially understood somatization as the unconscious and generally maladaptive amplification and exaggeration of physiological symptoms by the patient. In this regard, Kleinman shared the view of his medical and psychiatric colleagues of somatization as a fairly “primitive” mechanism, the inchoate expression of mental and social distress in the idiom of non-specific bodily complaints—complaints very much like, for example, *doenca de nervos* in Brazil. Somatization was seen as characteristic of marginalized, relatively powerless, psychologically unsophisticated and unreflexive, indeed mostly lower class and decidedly non-Western, men and women. I am thinking especially of Kleinman’s work on somatization in Chinese and Chinese Americans as “masked depression.”

Here I am suggesting, to the contrary, that humans everywhere, and *men* as well as *women*, employ their bodies in expressing complicated, contradictory, or hostile sentiments, especially when other avenues of expression are blocked or extremely dangerous. And so, negative sentiments may suddenly explode in an “epidemic” of seemingly “chaotic” and “unruly” bodily complaints and in symptoms that simultaneously breach and bridge the boundaries between mind and body, biology and culture, natural and social, individual, and collective.

We can look, for example, to the production of new, chronic, and annoying, if not disabling or life-threatening “diseases” in advanced post-industrial societies and among all social classes, of which the DSM-III(R) offers many illustrations. The “over-production” of disease has been the subject of much medical anthropological inquiry. Young (1989), for example, has studied the social and political meanings of post-traumatic stress in Vietnam War veterans. Martin (1987) has interpreted the “discovery” of pre-menstrual syndrome (PMS) as a medicalized discourse on the unacceptability of female irritability and barely suppressed rage, while Margaret Lock has documented the “invention” of the menopause and mid-life crisis in Japan as medicalized expressions of moral panic over the “modernization” of the Japanese family and gender roles. Lock and I have attempted to explain the phenomenon of the channeling of more and more personal dissatisfaction, longing, and protest into the idiom of sickness as a consequence, in part, of the secularization of social life and institutions where other forms of everyday resistance—such as gossip, witchcraft accusations, fantasy play, and the carnivalesque—have disappeared or lost their powers of enchantment.

On the other hand, it is easy to overlook the observation that people who live by and through their bodies in manual and wage labor, who live by their wit and by their grit, inhabit those bodies and experience them and *use* them in ways different from our own. The conventional psychiatric observation that poor and working-class people are particularly “prone” to somatization both diminishes and misunderstands an alternative and widespread form of body practice. The tendency in biomedicine, psychiatry, and even in conventional medical anthropology is to standardize our own socially constructed and culturally prescribed mind-body tactics and to label those that do not conform to these as deviant, pathological, irrational, psychologically primitive, or as inadequate. Against this view I wish to offer an interpretation of, for example, the embodied lives and somatic culture of the Nordestino sugarcane cutters (who I have been studying since 1982) as both normative and even as less “alienated” than the “psychologization” of social and political distress that *we* have unquestioningly accepted as a universal norm and standard. When personal and social distress is expressed psychologically rather than through a bodily idiom, the “natural” language of the body is suppressed, silenced and denied. I am suggesting here that the structure of individual and collective sentiments, right down to the feel of one’s body and the uses to which it is put, is an expression not only of cultural shaping, but also of one’s position and role in the technical and productive order.

When I refer, therefore, to the “somatic culture” of the displaced and marginalized sugarcane workers of the Alto do Cruzeiro, I mean to imply that theirs is a social class and culture that privileges the body and that instructs them in a close attention to the physical symptoms. Here I am following the lead of French phenomenologist, Boltanski (1984), who in his brilliant monograph translated into Portuguese as *Classes Sociais e O Corpo*, argues that somatic thinking and practice are commonly found among the working and popular classes who extract their basic subsistence from physical labor. He notes the tendency of the poor working classes in France to communicate with and through the body so that, by contrast, the body praxis of the bourgeois and technical classes may appear alienated and impoverished.

### Nervos in the Brazilian Northeast

Among the agricultural wage laborers living on the hillside shanty town of Alto do Cruzeiro, on the margins of a large interior market town in the plantation zone, who sell their labor for as little as a dollar a day, socioeconomic and political contradictions often take shape in the “natural” contradictions of sick and afflicted bodies. In addition to the expectable epidemics of parasitic and other infectious diseases, there are the more unpredictable explosions of unruly symptoms whose causes do not readily materialize under the microscope. I am referring to symptoms like those associated with *nervos* (frenzied nervousness)—trembling, fainting, and seizures, and paralysis of limbs—symptoms that disrespect and that breach mind and body, the individual, and social bodies.

These nervous attacks appear to be in part coded metaphors through which the workers express their politically dangerous and therefore unacceptable condition of chronic hunger and in part acts of defiance and dissent, graphically registering the afflicted one’s absolute refusal to endure what is, in fact, unendurable and their protest against the demand to work, meaning always in this context, the availability for physical exploitation and abuse at the foot of the sugarcane. And so rural workers who have cut cane since the age of seven or eight years and who today still earn no more than a dollar a day may occasionally collapse with a nervous crisis, or “*ataque de nervos*.” Their legs give way beneath them; they can no longer stand upright on their own two feet; they are left, metaphorically, without a leg to stand on.

In the exchange of meanings between the body personal and the social body, the nervous-hungry, nervous-weak body of the cane cutter offers itself as both metaphor and metonym of the “nervous” social–political system and for the weak position of the rural worker in the current economic order. In “lying down” on the job, and in refusing to return to the work that has over-determined most of their child and adult lives, the workers’ body language could be seen as a form of surrender and as the language of defeat.

But one can also see a drama of mockery and refusal. For if the folk ailment *nervos* attacks the legs, it leaves the arms and hands intact and unparalyzed—free for less physically ruinous work. Consequently, otherwise healthy young men suffering from nervous attack, can and do press their legitimate claims as “sickmen” on their political “bosses” and various patrons to find them alternative work, explicitly “sitting-down” work. As it is employed in this context, *nervos* is another version of the “Italian strike” alluded to earlier.

But *nervos* is an expansive and polysemous folk concept. Women, too, suffer from *nervos*, both the “*nervos de trabalhar muito*” (the “overwork” nerves from which male cane cutters suffer) *and* the “*nervos de sofrer muito*” (sufferers’ nerves). Sufferers’ nerves attack those who have endured a recent, especially a violent, shock or tragedy. Widows and the mothers of husbands or sons abducted and politically “disappeared” are especially prone to the mute, enraged, white-knuckled shaking of sufferers’ nerves. Here, Taussig’s (1989) notion of the “Nervous System” as a generative metaphor linking the tensions of the anatomical nervous system with the chaos and anxiety of the irritable, unstable social system is useful. One could read the current “nervousness” of the people of the Alto—expressed in an epidemic of *nervoso*—as a response to the “nervous” social and political system just now emerging after 20 years of repressive, military rule, but slowly and incompletely, with many vestiges of the military state still in place. On the Alto do Cruzeiro the military presence is most often felt in the late-night knock on the front door, followed by the scuffle and the abduction of one’s husband or son.

The “epidemic,” then, of *sustos* (fright sickness), *pasmos* (paralytic shock), and of *nervos* signifies a general state of alarm, of panic. It is a way of expressing, metaphorically, the “state of things” when one must move back and forth between an acceptance of the given situation as “normal,” “expectable,” and “routine”—as normal and taken-for-granted as one’s hunger—and an awareness of the real state of emergency in which one is plunged (see Taussig, 1989). And so, the rural workers of the shanty town are thrown from time to time into a state of nervous agitation, or shock, crisis, of *nervoso*, especially following the incidence of sudden and arbitrary violence against their social class, as in the disappearances, murder-mutilations, and roundups of young men and boys of the Alto do Cruzeiro.

To raise one’s voice in active political protest is as impossible as it is wildly dangerous. To be silenced, however, is intolerable. One is a man; one is a woman, after all. Into “impossible” situations like these, the body, through the powerful language of unruly symptoms, can keep alive the perception that a real “state of emergency” exists by inscribing the panic in the shaking, agitated nervous-hungry bodies of shanty town residents. Here, nervous sickness is a form of resistance, one that is both effective, in that it “publicizes” the danger, the fright, the “abnormality of the normal,” and yet “safe” in that it does not expose one’s self or one’s family members to further reprisals. Who can suspect the sick and the weak, after all? And conversely, who would reduce this complex, somatic and political idiom to an insipid discourse on patient somatization?

## Biomedicine and Resistant Illness

In short, illness and illness metaphors are often coded messages in the bottle, tossed onto angry seas by the suffering and the aggrieved, in the hope that a passing navigator may be able to retrieve the bottle and decipher the hidden meanings, the SOS of its contents. The “rub” is, however, that whenever protest and resistance are expressed in the language of symptoms and in bodily complaint, there is the consequent danger of an individualized and medicalized response. This is why I said that somatized illness, while one of the most common, everyday forms of resistance, is also one of the most problematic.

And so, the “negative” expression of their somatic culture concerns the tendency of these same exploited and exhausted Brazilian cane cutters to sometimes blame their situation, their daily problems of basic survival, on their own bodies, bodies that have seemingly collapsed and given way on them. A man will slap at his wasted or emaciated limbs (as though they were detachable appendages from the self) and say that they are “weak” and completely “useless.” A woman will pull at her breasts and a man will clutch his genitals and declare them “finished up,” “sucked dry.” Consequently, the people of the Alto look for strong, powerfully acting medications, drugs that will re-invigorate the body, “animate” the senses, and “fortify the bones.”

The danger of expressing personal anger, frustration, dissatisfaction, as well as social and political contradictions through the idiom of the body (even when invoking traditional, folk rhetorical devices such as “*nervos*”) is the possibility of their being absorbed by medicine and translated into a biomedical vocabulary *and* treated as a “disease” alone. Broad, flexible, socially constructed afflictions like *nervos* (or, for that matter like *susto*, *solidao*, or *pasmo* in Brazil or “School Refusal Syndrome” in Japan, or spirit attack in Malaysian factories, or “PMS” or worker “burnout” in the USA), while potent expressions of anger, defiance, and dissent, also create a space for their “medicalization” and “domestication.” When this happens the social and political implications of the afflictions are concealed from view and with them the possibility of using bodily distress to generate a radical critique.

### Medicalization: Cooptation of Illness

In this regard, the semantic confusion produced by an imperfect translation of the popular-to-biomedical stress model was useful; for example, to the Federal Aviation Administration (FAA) in its historic dispute with PATCO, the Professional Air Traffic Controllers Union in 1981. Union representatives had argued that air traffic control work, as it was presently organized, was unduly stressful on workers. The Union representatives meant, of course, that the demands made on air traffic controllers were unfair and intolerable, and that the workers were feeling overwhelmed, filled with tension, in short, “stressed out.” The FAA, however, cited a scientific study of a sample of air traffic controllers which showed no evidence of increased serum stress hormone levels in the workers. This was taken as incontrovertible evidence that air traffic control work was not overly stressful and it implied “malingering” on the part of PATCO strikers. The inability of the labor negotiators to grasp the problem caused by “mixed metaphors” and their deference to the scientific findings made them unable to prevent the FAA’s dismissal of the workers’ complaints and led to the mass firing of the strikers (Tesh, 1984).

So, too, Ong (1987) notes that the managerial and corporate view of the micro-electronics factory in Malaysia, buttressed by Western psychiatric models of hysterical disorders, is all too ready to convert dissident female workers into psychiatric “cases.” Both psychiatric and religious specialists are called into the factory and the “spirits of resistance” are dispersed, to be replaced, one is tempted to say, by the spirit of capitalism. The hegemonic tendencies of biomedical knowledge and practice that invite all manner of somatic complaint to be reinterpreted in light of its universalizing, essentializing discourse can signal the defeat of traditional idioms of resistance and noncompliance.

So, too, I have seen the metaphorization of hunger and child malnutrition, as one form of “*nervos*,” in the clinics and pharmacies of Northeast Brazil where talk about hunger can be seen as subversive and therefore as dangerous (Scheper-Hughes, 1988b). By the time that nervous-hunger reaches the *clinic*, the sufferer is already defeated. It is a sure bet that her suffering will be tranquilized so that medicine, even more than religion, comes to actualize the Marxist platitude on the drugging of the masses. There is no easy solution to the horns of this dilemma; it represents a classical double bind of sorts.

### The “sick role”

I do not wish to leave you with the impression that all forms of illness may be viewed in this light. I am perfectly willing to concede with the Gnau of New Guinea that “some illnesses just come,” that sometimes people are “sick nothingly,” that sometimes people just die, “by no purpose or intent” (Lewis, 1975). But whatever else illness is, and it is many things—an unfortunate brush with nature, a fall from grace, a social rupture, a cosmic disequilibrium—it is also, at times, an act of refusal. It can express itself in various ways: as a refusal to work or to struggle under oppressive and self-defeating conditions, and as a refusal to endure what is not, in fact, endurable, and a refusal to “cope.”

Assuming the sick role can communicate that one will *not* or simply *cannot* any longer. It is the strategy of Bartleby the Scrivener who “prefers not to.” This is the case with the nervous collapse of those “paralyzed” cane cutters who simply had had enough and had reached the end of their rope.

As Parsons (1972) recognized in his early and classic analysis of the sick role and society, sickness can pose a real threat to the stability of the social and moral order. Assuming the sick role can be a passive form of resistance, or, as I would say, a critical weapon of the weak. Parsons recognized the destructive and eroding effects of modern, industrial relations of production on the human spirit and body, and he understood (similar to the way Marx viewed religious behavior), that the over-production of diffuse bodily symptoms and complaints among industrial workers was a “sigh of the oppressed,” an expression of their frustration. Medicine offered an escape to the oppressed in the “lure and haven” of the “sick role,” “disability pay,” and “sick leave,” all of which Parsons viewed as forms of “sanctioned deviancy.” But these covert functions of the sick role were risky, and sickness had to be carefully monitored lest a “sickness strike” spread like wildfire among dissatisfied and disaffected people in society at large.

Hence, Parsons recognized one of the “hidden functions” of medicine in society at large was the control of the amount of discontent expressed in illness, and the diffusion of its radical potential. It was essential, therefore, that the physician fail to see the “secret indignation of the sick” (as Kim Hopper, 1982, once put it) and to transform fluid signs and symptoms of bodily protest into passive, individualized forms of breakdown, of illness and distress into reified and bona fide biomedical “diseases.”

### Listening to Illness

The sufferer of *nervos*, PMS, school refusal, worker stress and “burnout,” and so on has two choices. She can be open to the secret language of the organs that contains within it the seeds for critical reflection and *conscientizacao* (critical consciousness). “My *nervos*,” offered one Alto woman who had recently suffered the abduction-mutilation of her adult son, “is really just my life.” And another shanty town resident reached a similar conclusion: “My sickness,” she said, “is both physical *and* moral.”

On the other hand, the sufferer can silence the bodily scream of protest, cut it off by relegating more and more of her consciousness and pain to the technical domain of medicine where anger, frustration, anxiety, fear, and panic as well as nervous-hunger will be transformed into “diseases” to be treated with injections, “nerve pills,” hormones, and soporifics. Once “safely” medicated, the message in the bottle, that desperate and socially significant SOS is forever lost. The radical and reflexive moment has passed.

And so, the debate as to how cultural categories of distress can best be subsumed under biomedical categories of diseases becomes a “red herring” in the critical approach to illness that I am trying to foster here. The transformation of a culturally rich form of human communication into the individualizing language of physiology, psychology, or psychiatry is simply inappropriate.

What is crucially important for the medical anthropologist is to show how such polysemic terms as *nervos*, spirit possession, and stress, and the language and practice of trance, religious ritual, carnival play, and so on can be used in bringing to consciousness the links between the political and the social orders on one side and personal and physical distress on the other. If this form of communication that keeps the body metaphorically linked to mind and society is reduced to the “truthful” language of science, then one of the most impressive “weapons of the weak” is made unavailable in the struggle for relief from oppression.

## Conclusion

In conclusion, it is hoped that an awareness of the social and political relations that underlie some problematic expressions of illness (especially those illnesses conventionally understood as “somatization”) will give physicians, in the slightly altered words of the Alcoholics Anonymous credo, the courage to fix the things that can be fixed, the humility to recognize problems that fall beyond their sphere of knowledge and competence, and the wisdom to know the difference.

As for ourselves, our primary responsibilities and loyalties as critical anthropologists are with the *public* that increasingly sick-deviant, and alienated majority with whom we cast our lots. We need to remind the sick, the dying, and the stigmatized—whether they are the victims of AIDS or cancer, schizophrenia or PMS, heart disease or heartbreak, *susto* or *nervios*, depression or *solidao*, obesity or anorexia nervosa—that not everything that is biological is a disease, and not everything that is an illness is best managed solely within a biomedical framework.

In addition to chemotherapy is the political therapy of class action suits against the industrial and chemical polluters of our environment. In addition to “self-help” and “self-care” ideologies and therapies is the healthy dynamics of radicalized collectivities, subcultures for the dispossessed and angry sick such as the Boston Mental Patients Liberation Front and San Francisco’s chapter of ACT-UP. In the limited space of one’s own death sentence the mortally sick can supplement acts of individual self-preservation with existential acts of world-preservation, world-repair. We desperately need your help and your voices. To the anxious, the “over-stressed,” and the sad, we would suggest that in addition to psychotherapy, biofeedback, and stress management, there is union organizing and active protest—strikes, sit-ins, passive resistance—against the demands to endure what is, in fact, unendurable.

While it is essential to strip away the social stigmas that attach to disease, disability, and death (to which extent I agree with Sontag, 1978) we must be careful not to strip away the social content of human suffering. By interpreting illness, as I have done here, both as a biological and a social fact, and symptoms as a cryptic language of protest, we can readily discern the. sources of frustration, inequality, and oppression that are paradoxically hidden from view under the penetrating gaze of the X-ray, the ECG, and the CAT scan that while turning the body inside out, can severe body from mind and spirit.

The presence of suffering—especially when it is forced to express itself in the language of illness—exposes the gap between bodies that refuse the demand to suffer quietly, and the requirements of antagonistic economic and social orders. The task of bridging the three bodies—individual, social, and political—remains the missing link in a critical discourse on suffering and illness. I like to think of medical anthropology as providing the key to the development of a new epistemology and metaphysics of the body and of the *social* sources of illness and healing.

## Data Availability

No datasets were generated or analyzed during the current study.

## References

[CR1] Babcock, B. A. (Ed.) (1978). *The reversible world*. Cornell University Press.

[CR2] Bakhtin, M. (1968). *Rabelais and his world* (H. Iswolsky, Trans. Russian). M.I.T. Press.

[CR3] Bateson, G. (1958). *Naven. The culture of the Latmul of New Guinea as revealed through a study of the “Naven” ceremonial*. Stanford University Press.

[CR4] Boltanski, L. (1984). How a social group objectified itself: “Cadres” in France, 1936–45. *Social Science Information,**23*(3), 469–491.

[CR5] Bourdieu, P. (1977). *Outline of a theory of practice*. Cambridge studies in social anthropology (Vol. 16). Cambridge University Press.

[CR6] Buchbinder, M. (2015). *All in your head: Making sense of pediatric pain*. University of California Press.

[CR7] Comaroff, J. (1985). *Body of power, spirit of resistance*. University of Chicago Press.

[CR8] DaMatta, R. (1983). An interpretation of carnival. *Sub/stance,**37/38*, 162–170.

[CR9] Davis, N. Z. (1975). *Society and culture in early modern France*. Stanford University Press.

[CR10] Edel, L. (Ed.). (1964). *The diary of Alice James*. Dodd, Mead and Company.

[CR11] Eisenberg, L. (1977). Illness and disease. *Culture, Medicine and Psychiatry,**1*(1), 9–23. 756356 10.1007/BF00114808

[CR13] Favret-Saada, J. (1977). *Deadly words: Witchcraft in the Bocage*. Cambridge University Press.

[CR12] Favret-Saada, J. (1989). Unbewitching as therapy. *American Ethnologist,**16*(1), 40–56.

[CR14] Frank, G. (1986). On embodiment: A case study of congenital limb deficiency in American culture. *Culture, Medicine and Psychiatry,**10*(3), 189–219. 3757538 10.1007/BF00114696

[CR100] Führer, AG. & Vorhölter, J. (2025). Liberation Medicine: Past, Present, and Future. *Culture, Medicine and Psychiatry,**49*, 971–984. 41099927 10.1007/s11013-025-09949-w PMC12745312

[CR15] Gluckman, M. (1955). *Rituals of rebellion in South-East Africa*. Manchester University Press.

[CR16] Hamdy, S. (2008). When the state and your kidneys fail: Political etiologies in an Egyptian dialysis ward. *American Ethnologist,**35*(4), 553–569.

[CR17] Hobsbawm, E. (1965). *Primitive rebels: Studies in the archaic forms of social movement in the 19th and 20th centuries*. W.W. Norton.

[CR18] Hopper, K. (1982). *Paper read and discussed at the 81st annual meeting of the American Anthropological Association, Washington, DC in the session, “The Lure and Haven of Chronic Illness,” organized by Sue Estroff*.

[CR19] Illich, I. (1982). *Gender*. Pantheon Books.

[CR20] Kleinman, A. (1980). *Patients and healers in the context of culture*. University of California Press.

[CR21] Ladurie, L. (1980). *Carnival in Romans*. George Brazillar, Inc.

[CR22] Lewis, G. (1975). *Knowledge of illness in a Sepik Society*. Humanities and Athlone Press.

[CR23] Lewis, I. M. (1971). *Ecstatic religion: An anthropological study of spirit possession and Shamanism*. Penguin.

[CR24] Lock, M. (1986). Plea for acceptance: School refusal syndrome in Japan. *Social Science and Medicine,**22*(2), 99–112. 3749977 10.1016/0277-9536(86)90359-x

[CR25] Marriot, M. (1966). The feast of love. In *Krishna: Myths, rights and attitudes*. East-West Center Press.

[CR26] Martin, E. (1987). *The woman in the body: A cultural analysis of reproduction.* Beacon Press.

[CR27] Mauss, M. (1938). The notion of the person, the notion of the self. In M. Carrithers, S. Collins, & S. Lukes (Eds.), *Category of the person: Anthropology, philosophy, and history* (pp. 1–25). Cambridge University Press.

[CR28] McGranahan, C. (2016). Theorizing refusal: An introduction. *Cultural Anthropology,**31*(3), 319–325.

[CR29] Mullings, L. (1984). *Therapy, ideology and social change*. University of California Press.

[CR30] Ong, A. (1987). *Spirits of resistance and capitalist discipline*. State University of New York Press.

[CR31] Parker, R. (1990). *Bodies, pleasures and passions: Sexual culture in contemporary Brazil.* Beacon Press.

[CR32] Parsons, T. (1972). Definitions of health and illness in light of American values and social structure. In E. Gartley Jaco (Ed.), *Patients, physicians and illness* (2nd ed., pp. 107–127). Free Press.

[CR33] Rose Hunt, N. (2016). *A nervous state: Violence, remedies, and reverie in Colonial Congo*. Duke University Press.

[CR39] Scheper-Hughes, N. (1988a). ‘Carnival Marginal’: A topsy-turvy view of the Brazilian festival. In *Paper and videotape presented at the meetings of the American Anthropological Association*, Phoenix, Arizona, December 1988.

[CR34] Scheper-Hughes, N. (1988b). The madness of hunger: Sickness, delirium and human needs. *Culture, Medicine and Psychiatry,**12*, 429–458. 3219877 10.1007/BF00054497

[CR35] Scheper-Hughes, N. (1991). The rebel body. The subversive meanings of illness. *Traditional Acupuncture Society Journal,**10*, 3–10.

[CR36] Scheper-Hughes, N., & Lock, M. (1986). Speaking truth to illness. *Medical Anthropology Quarterly,**17*(5), 1-37–40.

[CR37] Scheper-Hughes, N., & Lock, M. (1987a). The mindful body: A prolegomenon to future work in medical anthropology. *Medical Anthropology Quarterly (New Series),**1*(1), 6–41.

[CR38] Scheper-Hughes, N., & Lock, M. (1987b). The mindful body: A prolegomenon to future work in medical anthropology. *Medical Anthropology Quarterly,**1*(1), 6–41. 10.1525/maq.1987.1.1.02a00020

[CR40] Scheper-Hughes, N., & Lock, M. (1990). Rituals and routines of discipline and dissent: Toward a critically interpretive medical anthropology. In T. Johnson & C. Sargent (Eds.), *Medical anthropology: A handbook of theory and method*. Greenwood Press.

[CR41] Scott, J. (1985). *Weapons of the weak: Everyday forms of peasant resistance*. Yale University Press.

[CR42] Simpson, A. (2014). *Mohawk interruptus: Political life across the borders of settler states*. Duke University Press.

[CR43] Sontag, S. (1978). *Illness as metaphor*. Farrar, Straus and Giroux.

[CR44] Strouse, J. (1980). *Alice James: A biography*. New York Review Books.

[CR45] Taussig, M. (1980). Reification and consciousness of the patient. *Social Science and Medicine,**14B*, 3–13. 7394562 10.1016/0160-7987(80)90035-6

[CR46] Taussig, M. (1989). Terror as usual: Walter Benjamin’s theory of history as a state of siege. *Social Text,**23*, 3–20.

[CR47] Tesh, S. (1984). The politics of stress: The case of air traffic controllers. *International Journal of Health Services,**14*(4), 569–587. 6500785 10.2190/JH2E-F62P-WMX8-7NQF

[CR49] Yates-Doerr, E. (2017). Counting bodies? On future engagements with science studies in medical anthropology. *Anthropology and Medicine,**24*(2), 142–158. 10.1080/13648470.2017.1317194 28721738 10.1080/13648470.2017.1317194

[CR50] Young, A. (1989). The moral order of a psychiatric unit treating war related posttraumatic stress disorder. In *Paper presented at the American Anthropological Association meetings*, Washington, DC, 1989.

